# Medical Students’ perceptions and attitudes toward Medical Leadership and Management

**DOI:** 10.12669/pjms.37.1.2406

**Published:** 2021

**Authors:** Sami Hamdan Alzahrani, Mukhtiar Baig, Anoud R. Omer, Mohammed R. Algethami

**Affiliations:** 1 Dr. Sami Hamdan Alzahrani, SBFM, ABFM. Assistant Professor, Consultant, Department of Family Medicine, Faculty of Medicine, King Abdulaziz University, Jeddah, Saudi Arabia; 2 Dr. Mukhtiar Baig, PhD. Department of Clinical Biochemistry/Medical Education, Faculty of Medicine, Rabigh, King Abdulaziz University, Jeddah, Saudi Arabia; 3 Dr. Anoud Rashad Omer, MD. Clinical Research Unit, King Abdulaziz University Hospital, Jeddah, Saudi Arabia; 4 Dr. Mohammed Ridha Algethami, MD. Preventive Medicine Resident, Joint Program Ministry of Health, Saudi Arabia

**Keywords:** Medical Leadership, Medical Management, Attitude, Perception

## Abstract

**Objectives:**

To find out medical students’ perceptions and attitudes toward medical leadership and management (MLM).

**Methods:**

A total of 336 medical students from the 2^nd^ to 6^th^ academic years from King Abdulaziz University (KAU), Jeddah, Kingdom of Saudi Arabia (KSA), were included in this cross-sectional study. This study was conducted in January-February 2020. The students were asked about their perceptions, attitudes, and interests in the leadership of medical care and clinicians. A four-part questionnaire was used for collecting data. SPSS-21 was used for analysis.

**Results:**

The participants included 172 (51.2%) males and 164 (48.8%) females. In total, 105 (31.3%) participants agreed that they had been very well educated about their perception, behavior, and interest in the field of medical leadership and clinic management, and 175 (52.1%) students agreed that clinicians should influence management decisions in a healthcare setting. Overall, 167 (49.7%) students agreed that management/leadership skills are important for clinicians. In total, 145 students (43.2%) desired to have more leadership training in medical school, and 129 (38.4%) students agreed to seek additional leadership/management training in their postgraduate research studies. When asked about their self-perception of good leadership skills, the students indicated that good leadership skills included integrity (47.9%), conflict resolution (46.7%), organization (44.4%), confidence (41.9%), communication (40.5%), self-reflection (40.2%), time management (33.6%), the ability to motivate others (36.9%), and the ability to keep calm under stress (33.3%).

**Conclusion:**

Many students were well aware of the MLM concepts. However, students agreed that management/leadership skills are important for clinicians, and there should be more leadership training in medical schools.

## INTRODUCTION

Professional growth in the medical field is based on clinical skills and medical expertise, which can be incorporated by introducing a competency-based curriculum in medical schools. An established curriculum should include leadership and management as one of the component frameworks designed to build up health care professionals. The current recruitment policies regarding medical experts should also focus on leadership qualities so that newly inducted physicians can cope with the advanced queries of the health care system. All doctors should be well oriented in their professional as well as moral and ethical responsibilities.^1^ Maintaining and upgrading current knowledge about their respective disciplines are always placed as the priority. However, the dynamic involvement of professionals as leaders can also provide beneficial connections in the management of patients, thereby establishing a high quality of patient care. Medical management calls for a direction to be set that will inspire and motivate other health professionals. In general, more clinicians have taken on the role of healthcare management to close the growing gap between clinicians and administrators.^2^

Healthcare is a diverse, complicated, and unpredictable field that is often better conducted by clinicians who have direct practical experience in patient care.^3^ Growing evidence now indicates that managerial decisions are being made by clinicians in whom managerial and leadership skills are commonly present.^4^ Clinical expertise and hospital performance have shown a strong association and have improved the financial, quality of care, and social performance in hospitals.^5^ In addition to their clinical training and obligations, health care providers should require clinicians to obtain leadership and management qualities to provide the best health care.^6^ Moreover, medical leadership has always depended on technical and academic skills, but often at the cost of so-called ‘softer’ characteristics, such as powerful emotional intelligence and excellent leadership. The current focus and initiatives are more effective than those of the past regarding medical leadership and management.^7^

Medical management and leadership are important skills to be acquired by physicians to enhance medical institution services and to provide high-quality care. Therefore, the results of this study will provide information regarding students’ knowledge and perception of medical management and address the need for leadership programs. This study’s objective was to identify the perceptions and attitudes of medical students toward medical leadership and management.

## METHODS

This cross-sectional, observational study was carried out on medical students in their 2^nd^ to 6^th^ academic years at King Abdulaziz University (KAU), Jeddah, KSA. This study was completed in January-February 2020. Ethical approval was obtained from the Research Ethics Committee of KAU (Reference No.22-3-20), and 336 medical students from various medical classes were enrolled in the study. Students were asked questions about their perceptions, attitudes, and interests in the leadership of medical care and clinicians. The questionnaire was developed from an already published study after the authors’ permission^8^ and consisted of four parts. The first part was demographic data, including gender, nationality, academic year, and GPA. In the nationality category, students were classified as Saudis or non-Saudis, and the GPA was classified into three categories of 1.5–3.49, 3.5–4.49, and above 4.5. The second part was related to students’ perceptions, attitudes, and interest in medical leadership and clinic management. The third part was related to the students’ self-perception of their leadership skills as identified by the Medical Leadership Competency Framework (MLCF).^9^ The fourth part was related to the students’ perception of their leadership training at KAU. Students answered questions on a 5-point Likert scale with choices of strongly agree, agree, neutral, disagree, and strongly disagree. Participants also rated their leadership skills as very good, good, satisfactory, poor, and very poor. Written informed consent was obtained from all participants, and they were informed about the nature of the study and the confidentiality of their responses.

### Statistical Analysis

SPSS version 21 was used to analyze the data. The descriptive statistics were presented in the form of frequencies and percentages for qualitative variables. The chi-square test was applied for qualitative categorical variables, and a p-value ≤ 0.05 was considered statistically significant.

## RESULTS

Among the 336 medical students enrolled in this study, 172 (51.2%) were males and 164 (48.8%) were females. Most students 322 (95.8%) were Saudis, and only 14 (4.2%) were non-Saudis. Students characteristics are shown in Table-I.

**Table-I:** Students’ characteristics.

Student Characteristics	Total N (%)
*Gender*	
Male	172 (51.2%)
Female	164 (48.8%)
*Nationality*	
Saudi	322 (95.8%)
Non-Saudi	14 (4.2%)
*Academic Year*	
2^nd^	81 (24.1%)
3^rd^	80 (23.8%)
4^th^	62 (18.5%)
5^th^	62 (18.5%)
6^th^	51 (15.1%)
*GPA*	
2.5 – 3.49	17 (5.1%)
3.5 – 4.49	151 (44.9%)
More than 4.5	168 (50.0%)

Students’ perceptions, attitudes, and interest in medical leadership and clinician managers were variable and are given in Table-II.

**Table-II:** Students’ perceptions, attitudes, and interest in medical leadership and clinician managers.

How strongly do you agree with the following statements regarding leadership training and opportunities?	Student Response N (%)				

	Strongly Agree	Agree	Neither Agree nor Disagree	Disagree	Strongly Disagree
“I am well informed about what a managerial position in medicine entails”	33 (9.8%)	105 (31.3%)	81 (24.1%)	97 (28.9%)	20 (5.9%)
“I think managerial decisions within a clinical setting should be influenced by clinicians”	77 (22.9%)	175 (52.1%)	66 (19.6%)	16 (4.8%)	2 (0.6%)
“I think it is important for clinicians to have managerial/leadership responsibilities”	122 (36.3%)	167 (49.7%)	28 (8.3%)	15 (4.5%)	4 (1.2%)
“I would like to have had more leadership training during medical school”	120 (35.7%)	145 (43.2%)	39 (11.6%)	26 (7.7%)	6 (1.8%)
“I would seek addition leadership/management training in my postgraduate studies”	87 (25.9%)	129 (38.4%)	76 (22.6%)	38 (11.3%)	6 (1.8%)
“I am interested in taking on leadership/managerial responsibilities during my career”	105 (31.2%)	132 (39.3%)	59 (17.6%)	34 (10.1%)	6 (1.8%)
“I think clinicians managerial/leadership opportunities should be highlighted and promoted to medical students”	106 (31.5%)	140 (41.7%)	76 (22.6%)	9 (2.7%)	5 (1.5%)

**Fig.1:**
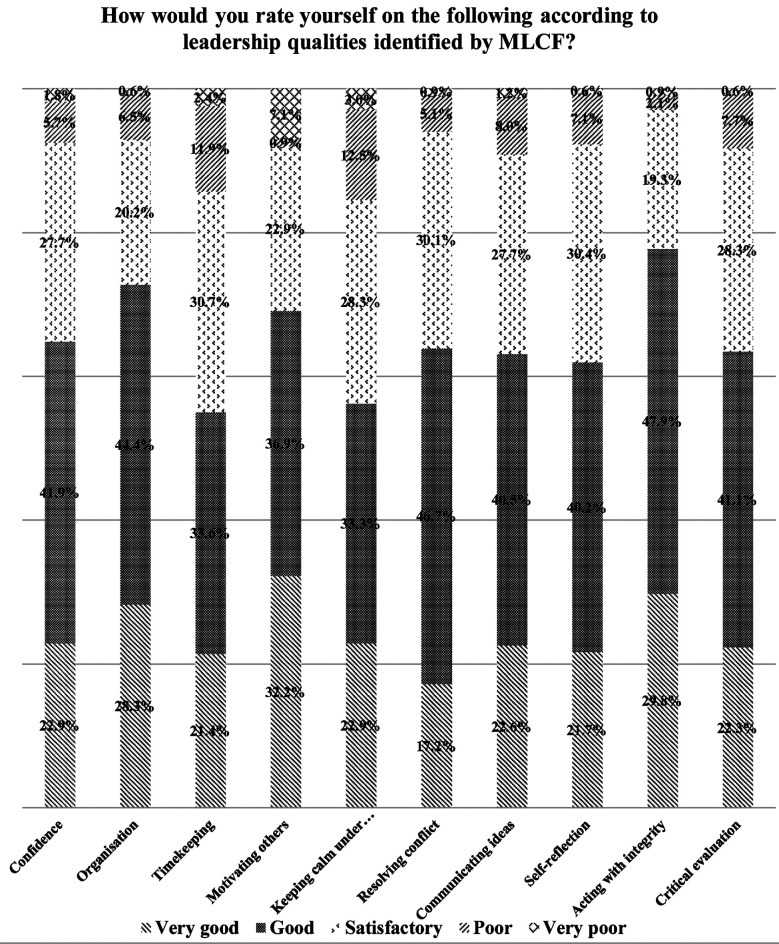
Students’ self-perception of leadership skills. MLCF= Medical Leadership Competency Framework.

Students self-perception of leadership skills according to Medical Leadership Competency Framework (MLCF) are presented in Fig.1.

The students’ perception of leadership training and experience in the medical college at KAU is depicted in Fig-II.

**Fig.2:**
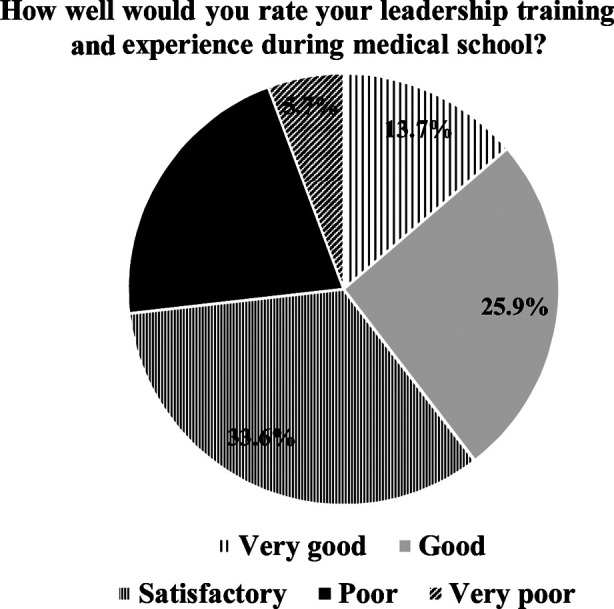
Students’ perceptions on Leadership training in King Abdulaziz University.

## DISCUSSION

As a subject, leadership has often been articulated in other fields as ‘professionalism’ or ‘communication skills,’ but efforts to create and evaluate leadership have been inconsistent and unstructured.^10^,^11^

Among the participants enrolled in our study, the majority strongly agreed or agreed that they were well informed about the medical managerial position. The majority also agreed or strongly agreed that clinicians should be responsible for the management policies within a healthcare setting, rather than an administrator who is not a clinician. Therefore, the students deemed it vital that a clinician possesses managerial/leadership skills. Most students indicated a desire to gain more leadership training during their studies. This study is the first of its kind to be carried out in KSA to evaluate medical students’ attitudes and opinions toward medical leadership and the idea of “clinical managers,” as suggested by the MLCF. The MLCF recommends that administration and leadership skills be achieved during the study tenure of medical students.

A previous study showed that medical students were interested in developing management and leadership ability and that 36% agreed strongly with encouraging medical students in these fields.^12^ More than 63% of the students believed they wanted more leadership training at medical schools, and more than half of the students evaluated their experience in management training in health care schools as bad.^12^ In a recent study, Quince et al. pointed out a need for clinical leadership and management training and a necessity for students to understand healthcare professionals’ broad viewpoints. The study suggested that the majority of the students, if not all, indicated a desire for the incorporation of medical leadership and management programs into undergraduate medical curricula.^13^ A British study reported that the two-thirds of the medical schools and almost all the students (93%) appraised the importance of teaching MLM topics.^14^

In accordance with our results, Wong et al. reported that the state of quality care in medical setups was directly linked to medical education in fields related to MLM and that this education not only improved the quality care of patients but also prevented the occurrence of avoidable errors.^15^ A systematic review of leadership and management attitudes of medical students reported a desire among medical students for more leadership and management training. However, a shortage of time due to competing for instructional needs and potential lack of participation on the part of some students and faculty in the activity were few possible obstacles to the adoption of MLM in health care schools.^16^ Our study suggests that the desire for further education in these fields continues to be strong among more contemporary medical students.

Hadley et al. implied that while MLCF was introduced in the United Kingdom, leadership education for training doctors is still in its early stages. They further proposed that clinicians should also assess leadership competencies beside clinical skills, and proper feedback should be provided to students.^17^ Another approach for encouraging medical school leadership training is by offering a formal management course; this has proved helpful and had a powerful satisfaction score among postgraduate medical students.^18^ Our study participants evaluated themselves high on the ten specific features outlined by the MLCF requirements, despite the inadequate MLM training at medical college. This result is corroborated with Rouhani et al. (2018).^8^ In a UK-based study, almost all the students (95 %) had received little or no training in MLM, and when asked whether these topics should be taught formally as part of their medical school curriculum, almost all indicated ‘yes’.^19^ This shows that even at a medical college in the UK, little focus is placed on teaching medical leadership or management, indicating the low level of importance given to the subject.

When leadership in hospitals and practice is compromised, the result is often a decline in patient care.^20^ Independent investigations of failed hospitals have previously shown that a lack of leadership and management was the cause of continued adverse patient care and outcomes.^21^ Consistent delivery of high-quality care to patients is among many other variables that depend on top medical leadership. 22

A systematic review reported that fresh graduates recognize “leadership as individualistic and hierarchical” and are only marginally prepared to fulfill this position.^23^ Al-Omari et al. (2020) stated that medical students should understand that leadership offers opportunities to improve things. Irrespective of their rank or title, medical students should understand that any position they have has the potential for leadership. Anyone can influence and make a difference in their surroundings. They further suggested that faculty and curricula should work to enhance the vision of leadership.^24^

MLM is an essential ability. Students need to be able to make judicious management choices in real-life practice. MLM’s introduction for today’s students would help them, as doctors of tomorrow, to solve the growing complexities of modern health care.^18^ Our study results are more or less comparable to almost all studies mentioned in the discussion. Several studies have shown overwhelmingly positive medical students’ attitude and want to learn more about MLM concepts. These studies also emphasized the need for management and leadership courses for medical students.^12^,^17^,^23^,^24^ Students’ good perceptions and optimistic attitudes have demonstrated that they are cognizant of MLM skills because of their growing need in their future careers. They perceive it as one of the essential skills for their future endeavors.

### Implications of the findings

The findings of our research have demonstrated that many medical students are well aware of MLM principles, and they acknowledged that management/leadership qualities are essential to physicians and that more leadership instruction should be offered in medical school. Authorities should promote medical leadership for young trainee physicians during their student life. There is a need to inculcate leadership skills among young physicians; therefore, we suggest a two-week short leadership module. For this module, blended learning (BL) can be employed, and renowned faculty members from the country and outside the country can be invited to teach. A recent study from KAU already emphasized BL utilization and importance.^25^ However, all studies, including ours, show that teaching of these subjects should be encouraged to improve the skills of the students and achieve overall improvements in health care management.

### Limitations of the study

Our study was limited to a single institute in KSA, so it does not represent the overall country’s medical student population. Selection and observer biases could have also played a part as limitations of the study.

## CONCLUSION

Many students were well aware of the MLM concepts. However, students agreed that management/leadership skills are important for clinicians and there should be more leadership training opportunities during their undergraduate training. Our results also suggest a small gap in Saudi medical schools’ quality of training regarding leadership and management skills. Further multi-institutional studies would provide a wider perspective regarding medical students’ opinions and would aid in guiding future prospects in terms of addressing medical leadership and management.

### Authors’ Contribution:

**SHA:** Designed the research, did statistical analysis, drafted the manuscript and responsible and accountable for the accuracy or integrity of the work. **MB:** Statistical analysis and drafted the manuscript. **ARO, MRA:** Contributed to data collection and analysis and manuscript writing.
